# What happens to ART-eligible patients who do not start ART? Dropout between screening and ART initiation: a cohort study in Karonga, Malawi

**DOI:** 10.1186/1471-2458-10-601

**Published:** 2010-10-12

**Authors:** Nuala McGrath, Judith R Glynn, Jacqueline Saul, Katharina Kranzer, Andreas Jahn, Frank Mwaungulu, Msenga HC Ngwira, Hazzie Mvula, Fipson Munthali, Venance Mwinuka, Lorren Mwaungulu, Paul EM Fine, Amelia C Crampin

**Affiliations:** 1 London School of Hygiene and Tropical Medicine, London, UK; 2 Africa Centre for Health and Population Studies, University of KwaZulu Natal, Mtubatuba, South Africa; 3 HIV Department, Ministry of Health, Lilongwe, Malawi; 4 Karonga Prevention Study, Chilumba, Malawi; 5 Karonga District Hospital, Karonga, Malawi

## Abstract

**Background:**

Routine ART programme statistics generally only provide information about individuals who start treatment. We aimed to investigate the outcome of those who are eligible but do not start ART in the Malawi programme, factors associated with this dropout, and reasons for not starting treatment, in a prospective cohort study.

**Methods:**

Individuals having a first screening visit at the ART clinic at Karonga District Hospital, northern Malawi, between September 2005 and July 2006 were interviewed. Study follow-up to identify treatment outcomes was conducted at the clinic and in the community. Logistic regression models were used to identify factors associated with dropout before ART initiation among participants identified as clinically eligible for ART.

**Results:**

88 participants eligible for ART at their first screening visit (out of 633, 13.9%) defaulted before starting ART. Participants with less education, difficulties in dressing, a more delayed ART initiation appointment, and mid-upper arm circumference (MUAC) < 22 cm were significantly less likely to have visited the clinic subsequently. Thirty-five (58%) of the 60 participants who defaulted and were tracked at home had died, 21 before their ART initiation appointment.

**Conclusions:**

MUAC and reported difficulties in dressing may provide useful screening indicators to identify sicker ART-eligible individuals at high risk of dropping out of the programme who might benefit from being brought back quickly or admitted to hospital for observation. Individuals with less education may need adapted health information at screening. Deaths of ART-eligible individuals occurring prior to ART initiation are not included in routine programme statistics. Considering all those who are eligible for ART as a denominator for programme indicators would help to highlight this vulnerable group, in order to identify new opportunities for further improving ART programmes.

## Background

In Malawi, adult HIV prevalence has stabilised at about 14% since the late 1990s[1]. Scale-up of antiretroviral treatment (ART) began in Malawi in 2004, with initial selection of 60 hospitals across the country to provide broad geographical coverage[2,3]. The Malawi national ART programme follows a public health model focusing on 'service delivery to all who need it'[4]. A generic, fixed-dose combination treatment (Triommune) with stavudine, lamivudine and nevirapine is available as first line treatment and given free of charge to eligible patients. An alternative first line treatment option is available if an individual has unacceptable side effects to Triommune. Second line treatment is available if an individual is considered to have failed first line treatment. According to national guidelines, individuals are eligible for ART in Malawi if, upon physical examination and history during a screening clinic visit, they were assessed to be in stage III/IV, or stage II with a CD4 count < 250 cells/mm^3^. A guardian/buddy is required to attend a group counseling session and treatment initiation with the eligible individual. This guardian is subsequently allowed to collect medicines monthly on behalf of the patient on condition that the patient attends the clinic every three months.

The ART clinic at Karonga District Hospital opened in June 2005, through a partnership between the Malawi Ministry of Health and Karonga Prevention Study. The clinic was assessed to be a 'medium burden' clinic by the Ministry of Health and allowed to enroll 50 new patients a month onto ART. A number of patients already on ART in other districts were also transferred in, most as soon as the clinic opened. Transfers-in were additional to the monthly target. The ART clinic was open for screening visits three days a week, with treatment visits on the other two days. Ideally the group counseling session occurred 2-3 days after staging, with ART initiation one week later. However, this varied depending on the length of the waiting list. Two appointments a week were kept 'open' to fast track individuals who were very sick but deemed stable enough to initiate immediately. No CD4 tests were available at the hospital laboratory. Therefore, eligibility for ART initiation in Karonga was determined using WHO stage. In this district, in common with most areas of Malawi, diagnostic facilities were also limited and it was not possible to obtain microbiological, histological or imaging support for WHO Stage 4 diagnoses. The alternative first line and second line treatment options were only available for the northern districts at Mzuzu hospital, approximately three hours by road from Karonga District Hospital.

The Karonga ART clinic used the simple standardised national documentation system designed by the National ART programme for the providers of ART in the public sector, to generate routine key statistics. This nationwide system has demonstrated that the programme has been successful in terms of retention and survival among those who start ART [5]. However, little is known in Malawi about the outcome of those who are eligible but do not make it into the programme[6] or their reasons for not starting treatment[7]. In this paper we measure the extent of loss of ART-eligible individuals between screening and ART initiation and identify risk factors associated with this loss.

## Methods

In addition to data available from the national documentation system, additional data were collected for this study in the screening clinic and at home visits. All patients attending a first screening visit at the Karonga ART clinic between September 2005 and July 2006 were eligible for inclusion in this study and were assigned a unique ID number independent of the government programme number series (which is only assigned to those who start ARVs). On recruitment into the study, all participants were asked to provide written consent for the additional data collection. During the screening visit, socio-demographic and clinical data were collected (see Additional file 1 for clinical questions) including: age, sex, occupation, marital status, level of education, village of residence, HIV stage, weight and height, mid-upper arm circumference (MUAC), and indicators for activities of daily living (dressing, washing, eating/drinking, toileting, walking 100 m).

A clinician staged each individual's HIV disease and, if ART-eligible, assessed their readiness to start treatment based on their understanding of the treatment benefits, willingness to adhere and to continue even if they felt better, and their ability to attend the clinic regularly (in terms of costs, that they lived in the district etc). Some individuals were considered ART-eligible, however they were too sick to start immediately and needed to be stabilized before starting ART. Others had medical contra-indications and were told to come back later; they were taking ketaconazole, or were in the first trimester of pregnancy, or were in the intensive phase of TB treatment (although this has since changed as a contra-indication). For individuals who were told that they were not yet clinically eligible on the first visit, clinicians would make appointments if they wanted to review the person again, otherwise they were asked to return when they had symptoms.

Follow-up of those who were identified as eligible for treatment but did not initiate ART was conducted between February and December 2006. Tracking was conducted by the same team that were following treatment defaulters from the Karonga ART clinic. Given limited resources, priority was given to tracking treatment defaulters which sometimes delayed/prevented the tracking of non-initiators. Deaths were reported by relatives to staff at the clinics or during tracking visits. Ethics approval was given by the Malawi National Health Sciences Research Committee and the London School of Hygiene & Tropical Medicine, UK.

### Statistical Analysis

All analyses were conducted in Stata 10.0 (Stata Corp., College Station, Texas, USA). Logistic regression models were used to identify factors associated with dropout before ART initiation among participants identified as clinically eligible for ART at their first screening visit. Age and education level were considered as categorical variables. Malawi's education system provides 8 years of primary and 4 years of secondary school. Students may study in any of the three major local languages for most of the first 4 years of primary school after which English becomes the medium. Thus, for our models we grouped individuals who had no schooling, those with 1-4 years of primary education, those with 5+ years of primary education but never attended secondary school, and those who attended at least one year of secondary or continued through further education. The distance between each participant's village and the ART clinic was calculated using GPS coordinates pre-recorded for each local village, and a group of indicators representing distance between residence and the ART clinic were considered in the models. Height and weight measures were used to calculate body mass index (BMI) and a binary indicator of chronic energy deficiency (CED, using a BMI cutoff of <18.5) was considered in the analyses. Mid-upper arm circumference (MUAC) was used to create a separate indicator of CED; (grade 1 or higher vs no CED) defined as MUAC < 22.0 cm for females and <23.0 cm for males. The questions asking about difficulties in daily living were based on self-care and mobility questions from the activities and participation domains section of the WHO International Classification of Functioning, Disability and Health[8]. Initially, each question was considered in the analysis using a separate indicator to represent functioning (any difficulty vs no difficulty) in a particular activity. In addition, a composite indicator was created to represent reported difficulty in any activity vs no reported difficulties. Indicators were used to represent four 3-month periods during study recruitment in order to investigate whether the characteristics of individuals at the time of first presentation changed with the length of time since the clinic opened (eg. with different WHO stage). A separate set of indicators was used to represent whether an ART initiation appointment was given to the ART-eligible individual for 0-7 days, 8-30 days or 31+ days after their screening visit. These categories were chosen to reflect the target of giving ART initiation appointments within a week of screening, vs a delay in the appointment date, and splitting that delay into less than or more than 1 month.

For the multivariate models, we used a stepwise procedure to determine the final model considering all variables significant in the univariate analyses, sex and age, and each separate indicator of daily functioning.

Among those not clinically eligible at first presentation, descriptive statistics were used to document how many returned for a second or third screening visit and how many were found to be ART-eligible at the later visits.

## Results

759 individuals attended the screening clinic for the first time during this period and 730 (96.2%) were interviewed and consented to participate. Twenty-two patients were missed by the study team during busy clinic sessions and seven refused. The median age of the participants at first presentation was 36.7 years, (IQR 30.9 - 44.3), with 9 <18 years old (median 14.9, range 13.3-15.8 years). Forty percent (40%) were male, 43% reported farming as their occupation, and 55% were assessed to be in WHO stage IV. Forty-eight percent (48%) were currently married and 28% were widowed. Only 27% had ever attended secondary school. Almost universally (>99%), participants reported having sought care within the last 6 months from a hospital/health centre or a traditional healer, a private practitioner or a combination of these.

**Figure 1:**
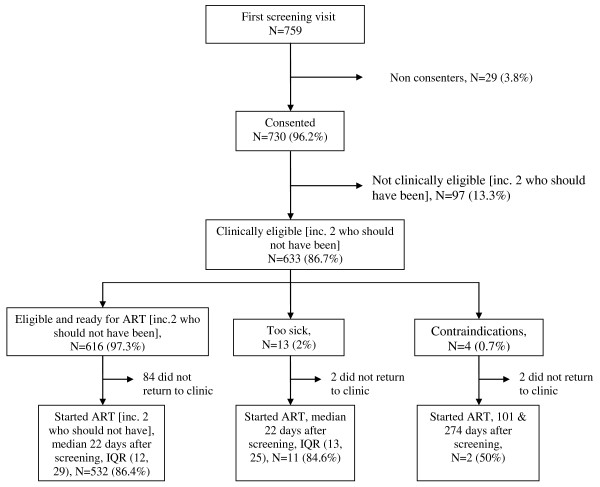
**Outcome of first screening visit and ART initiation status**.

Six hundred and thirty-three (633/730, 86.7%) were told they were clinically eligible for treatment (Figure 1), including six individuals who were in WHO stage II but had a CD4 count < 250 cells/mm^3 ^established through their participation in a research study. However, 17 of these participants were not ready to start treatment because they were too sick (N = 13) or had contra-indications (N = 4). Of the 616 participants identified as ready to start treatment, 532 (86.4%) did so, a median of 22 days (IQR 12-29 days) after the screening visit, with 10% starting after more than 40 days. In addition, 13 of the 17 clinically eligible participants initially advised not to start treatment started later (Figure 1), a median of 22 days (IQR 13-27 days) after the screening visit. This leaves 88 (13.9%) individuals who were clinically eligible for ART after the first visit, but who did not return to clinic. Table 1 shows the distribution of selected characteristics among those clinically eligible to start treatment at their first screening visit and odds ratio (OR) estimates of association between these factors and dropout before starting ART. In the final multivariate model, there was a significantly higher risk of dropout associated with lower education, grade 1 or higher chronic energy deficiency (CED), difficulty in dressing, a more delayed ART initiation appointment, and being screened in the later calendar periods of the study. A test for linear trend in calendar period was not statistically significant (χ^2 ^test for trend was 3.27 on 1 degree freedom, p = 0.07). There was no significant association with age, sex, current marital status, distance of residence from clinic, or WHO stage, even in univariate models. In a univariate model, BMI < 18.5 was significantly associated with a significantly higher odds of dropout, however this did not remain significant in the multivariate model that included an indicator of CED using MUAC. An alternative model to the final model considered the composite indicator of difficulty in any daily functioning instead of difficulty in dressing and found the composite indicator not to be significantly associated with the odds of dropout (likelihood ratio test p = 0.65).

**Table 1:** Univariate and multivariate logistic regression models for odds of dropout after first screening visit despite being clinically eligible to start ART1

Characteristic	N (% dropout)	Univariate Hazard Ratios^2^, 95% CI	Multivariate Hazard Ratios^2^, 95% CI
**Sex **: Female	369 (14)	1.0	
Male	263 (14)	0.99 (0.62, 1.57)	
**Age (yrs): < 30**	137 (14)	1.0	
30- <40	255 (15)	1.12 (0.62, 2.03)	
40+	240 (12)	0.85 (0.46, 1.59)	
**WHO stage **: IV	404 (13)	1.0	
II^3 ^or III	228 (15)	1.23 (0.77, 1.95)	
**Education**			
None	40 (30)	2.89 (1.36, 6.11)**	3.10 (1.40, 6.86)**
1-4 years Primary	85 (19)	1.56 (0.83, 2.94)	1.57 (0.81, 3.02)
5+ years Primary	325 (13)	1.0	1.0
Secondary or higher	174 (8)	0.59 (0.31, 1.11)	0.67 (0.35, 1.29)
Missing	8 (38)	4.04 (0.93, 17.5)	5.83 (1.21, 28.1)
**Occupation**			
Farmer	267 (17)	1.0*	
Other occupation	197 (9)	0.48 (0.27, 0.86)	
Not working	163 (14)	0.75 (0.43, 1.30)	
Missing	5 (20)	1.20 (0.13, 10.99)	
**Current marital status **(1 missing)			
Married	299 (13)	1.0	
Divorced	132 (14)	1.09 (0.60, 1.96)	
Widowed	171 (14)	1.06 (0.61, 1.82)	
Single	29 (14)	0.96 (0.34, 3.13)	
**Distance to clinic**			
<2 km	100 (13)	1.0	
2-5 km	148 (7)	0.54 (0.23, 1.25)	
> = 5 km	375 (14)	1.13 (0.59, 2.16)	
Missing	9 (100)	-	
**Body Mass Index**			
No CED	299 (10)	1.0*	
CED4	326 (17)	1.93 (1.20, 3.12)	
Missing	7 (29)	3.72 (0.69, 20.1)	
**MUAC**			
No CED CED5	349 (10) 283 (18)	1.0** 2.02 (1.27, 3.20)	1.0** 1.91 (1.17, 3.12)
**Difficulty in dressing**			
None	556 (13)	1.0*	1.0*
Slight/greater difficulty	76 (22)	2.00 (1.10, 3.63)	2.34 (1.22, 4.50)
**Difficulty in daily functioning^6^**			
None	251 (13)	1.0	
Slight/greater difficulty	381 (14)	1.09 (0.68, 1.74)	
**Period of first presentation**			
Sept -November 2005	124 (6)	1.0**	1.0*
Dec 2005 - Feb 2006	200 (17)	3.42 (1.47, 7.99)	3.81 (1.59, 9.15)
March - May 2006	224 (14)	2.68 (1.14, 6.29)	2.31 (0.94, 5.68)
≥ June 1, 2006	84 (18)	3.63 (1.41, 9.35)	2.48 (0.82, 7.52)
**Timing of ART initiation appt given relative to screening visit**			
0-7 days	81 (7)	1.0*	1.0**
8-30 days	439 (13)	1.90 (0.79, 4.57)	2.61 (1.03, 6.64)
31+ days	112 (21)	3.23 (1.25, 8.35)	5.04 (1.71, 14.8)

^1 ^N = 632 since mid-upper arm circumference was included in the final multivariate model and one individual was missing this variable (they were not seen again at the clinic).

^2 ^likelihood ratio test p value; *p < 0.05, **p < 0.01, ***p < 0.001

^3^Six had a low CD4 count recorded

^4 ^WHO recommends the use of BMI < 18.5 for grade 1 of higher chronic energy deficiency (CED).

^5 ^WHO recommends the use of sex specific MUAC cut off points of <22 cm for female and <23 cm for men for CED

^6 ^Any difficulty in washing or dressing oneself, toileting, eating/drinking, or walking 100 m w/out a stick, in response to the question "On an average day in the last week, how difficult has it been for you to perform the following activities without any kind of assistance at all?"

Forty-eight (49%) of the 97 individuals who were told that they were not yet clinically eligible on the first visit were invited by the clinician to return for a second screening, however the dates of these new screening visit appointments were not captured in the study database. Sixteen of these 48 returned for a second screening, a median of 61 days (IQR 25, 100) after their first screening. In contrast, 15 of the 49 participants asked to return when they had symptoms returned a median of 80 days (IQR (30, 173) after their first screening visit. Thus overall, 31 participants returned for a second screening, and 25 of them were eligible for ART. One of these 25 was too sick and one had contra-indications so were not started on ART immediately, and neither returned, and three other individuals did not return to the ART clinic. The remaining 20 started ART. Among the 6 who were not clinically eligible at their second visit to the screening clinic, 3 returned for a third visit at which time one was clinically eligible for ART and started treatment.

Combining the first, second and third screening visits, 93 eligible participants (out of 659, 14%) defaulted before starting ART. We tracked 60 (65%) of these at home, a median 55 days after they had missed their ART appointment (IQR: 35, 83). Thirty-five (58%) had died, 21 before their ART initiation appointment and the others soon after (median 19 days after appointment, IQR 7, 47). Three individuals had left the area and two could not be traced. Of the 20 individuals found alive at follow-up, 4 reported illness as the main reason for not attending, 8 reported lack of money for transport, 4 could not find a suitable guardian, 2 were too busy, one felt well after the screening visit, and one gave no reason. One of the individuals who reported ill health as the reason for not attending at the first follow-up visit, was found through a second home visit to have died soon after. Twenty one (64%) of the 33 not sought in the community had ART initiation appointments after mid-June 2006, i.e. shortly before the end of the study.

## Discussion

We show that in this ART programme 14% of individuals who would potentially have benefited from treatment did not start ART. This is similar to the estimate (16.4%) from a retrospective cohort study in South Africa[9] but lower than the 25.5% reported by a retrospective cohort study in southern Malawi[7]. Similar to the experience of TB programmes, exclusion from analysis of patients who die or are lost between presentation and starting treatment may potentially bias programme estimates of treatment outcomes, making them difficult to interpret and potentially misleading[10,11].

In a context where CD4 counts are not available, MUAC and reported difficulties in dressing may provide useful screening indicators to identify sicker ART-eligible individuals at high risk of dropping out of the programme who might benefit from being brought back quickly or admitted to hospital for observation. The finding that WHO stage IV was not associated with a higher odds of dropout before ART initiation compared to those in WHO stage III was unexpected. This may reflect the specific policy of keeping some appointments each week available for sicker patients, or represent an increased effort by individuals to seek treatment because they were sicker. Higher odds of defaulting among lower educated individuals has been shown elsewhere in Africa and may suggest that the content, amount and complexity of information given to patients during the screening visit may need to be adapted [12].

The significant positive association between dropout and length of delay from screening to the initiation appointment is consistent with a Cambodian study [13]. Now that the service at Karonga is well established, delays between screening and starting treatment are no longer a prominent feature. This is likely to change if criteria are altered to enable people to start treatment at an earlier stage, and may require a two stream service to ensure that those in more clinical need are not affected by waiting lists. The finding that participants enrolled in the later calendar periods of the study had a significantly higher odds of dropout compared to those recruited in the earliest period of the study may be due to particularly motivated patients coming for screening as soon as the clinic opened.

Previous work by our group has suggested that ART eligibility based on clinical staging criteria alone may miss up to two-thirds of those considered eligible using criteria based on clinical staging and CD4 cell count[14], and has highlighted a need for simpler CD4 testing methods. However, in countries with constrained resources, and increasing decentralisation of services, the current available technologies make it unlikely that CD4 testing will be available in small health centres that are now integral to ART programmes. Where equipment is available and CD4 testing is a policy, challenges remain in ensuring no interruptions in the supply of reagents, power supply and trained technicians. In many aspects the ART Clinic in Karonga operated like any other district ART clinic in Malawi, characteristic of the simplified public health approach established by the Malawi Ministry of Health[15], furthermore outcomes of those who started ART at Karonga[16] were not significantly different to those reported from other clinics[17-19]. The results of this study are therefore generalisable to other clinics in similar contexts.

We found that 58% of defaulters followed up in the community had died; 60% of whom died before their initiation appointment. This high level of pre-treatment mortality is consistent with findings from a South African study[20], and emphasize the need for priority initiation and improved availability of key drugs and clinical management. However, the proportion of individuals in WHO stage 4 at screening (55%), is higher than reported in established ART clinics in rural Malawi for the same period[21] and more recently[22]. Our study population included HIV positive people who may have been eligible for some time but had no local access to ART previously.

Among those found to be alive at the tracking visit, the most frequently reported barrier to returning to the clinic was cost of transport, a barrier that has also been documented in centralised prevention of mother-to-child transmission and ART programmes elsewhere in rural Malawi[23,24]. These reports suggest that targeted support may be beneficial at screening visits but poverty-related barriers are likely to be persistent and also affect long-term retention on ART. More recent devolvement of ART initiation from the district hospital alone to additional rural hospitals within the district, resulting in shorter distances for individuals to attend an ART clinic is likely to have eased this barrier.

Several other ART-eligible patients alive at the tracking visit cited lack of a suitable guardian/buddy as a barrier to ART. The policy of requiring a guardian to accompany individuals until they are established on ART remains part of the national programme in Malawi. This policy is based on experience of the national TB treatment programme and its impact has not been formally evaluated in the ART programme. In a society where literacy and education levels are low, a guardian also receives the treatment-related education and can support the individual, remind them to take drugs, help with drug taking, attend clinic on their behalf etc. In Malawi, hospital patients are expected to come with a guardian to provide basic nursing care - washing, feeding, toileting etc. In the ART programme context, guardians can also provide physical help to get to clinic, and care whilst at the clinic.

ART programme success is currently measured as the proportion continuing to receive ART among those who started treatment and survived. However we have shown that there are many patients who are considered eligible for ART, but do not start treatment. For many of these patients the reason for not receiving ART was that they had died. These early deaths are not included in routine programme statistics. Considering all those who are eligible for ART as a denominator for programme indicators would help to highlight this vulnerable group, in order to identify new opportunities for further improving ART programmes.

## Competing interests

The authors declare that they have no competing interests.

## Authors' contributions

This paper was written by NM, JRG, KK, AJ, PEMF and ACC. NM, JRG, JS, KK, AJ, FrM and ACC contributed to the conception and design of the study. NM, JS, KK, AJ, FrM, MN, HM, FiM, VM, LM and ACC acquired data or actively participated in data management activities. NM performed the statistical analysis. NM, JRG and ACC drafted the report, and all co-authors (except for the late FrM) revised the manuscript. NM, AJ, FrM and ACC, supported by JRG and PEMF, obtained funding for the study.

## Pre-publication history

The pre-publication history for this paper can be accessed here:

http://www.biomedcentral.com/1471-2458/10/601/prepub

## Supplementary Material

Additional file 1 **Appendix 1: Clinical questionnaire**. This questionnaire was used to collect clinical data at screening visits in the Karonga ART clinic. The section 'Findings' is a clinical tool/checklist of symptoms and signs that we developed in order to lead the clinician systematically through all the AIDS defining criteria when ascertaining ART eligibility. In Karonga district, in common with most areas of Malawi, diagnostic facilities are limited and it is not possible to obtain microbiological, histological or imaging support for WHO Stage 4 diagnoses. Click here for file
