# Supplementary material for: What happens to ART-eligible patients who do not start ART? Dropout between screening and ART initiation: a cohort study in Karonga, Malawi
Source: BMC Public Health. 2010 Oct 12;10:601. doi: 10.1186/1471-2458-10-601 (PMC2964626; doi:10.1186/1471-2458-10-601)
Supplement: Additional file 1 — Appendix 1: Clinical questionnaire. This questionnaire was used to collect clinical data at screening visits in the Karonga ART clinic. The section 'Findings' is a clinical tool/checklist of symptoms and signs that we developed in order to lead the clinician systematically through all the AIDS defining criteria when ascertaining ART eligibility. In Karonga district, in common with most areas of Malawi, diagnostic facilities are limited and it is not possible to obtain microbiological, histological or imaging support for WHO Stage 4 diagnoses. [file 1471-2458-10-601-S1.DOC]

|  |  | | | | | | | | | | | | | | |  | | | | | | | | | | | | |  | |
| --- | --- | --- | --- | --- | --- | --- | --- | --- | --- | --- | --- | --- | --- | --- | --- | --- | --- | --- | --- | --- | --- | --- | --- | --- | --- | --- | --- | --- | --- | --- |
|  | **On an average day in the last week, how difficult has it been for you to perform the following activities without any kind of assistance at** **all?** | | | | | | | | | | | | | | |  | | | | | | | | | | | | |  | |
|  | **0** | No difficulty | **1** | Slight difficulty | | **2** | Moderate difficulty | **3** | | Severe difficulty | | **4** | | Unable to carry out activity | |  | | | | | | | | | | | | |  | |
| 1 | Walking without stick from OPD to Buyu clinic (100m) | | | | | | | | | | | | | | | 0 | 1 | | | | 2 | | | | 3 | | | 4 | walking | |
| 2 | Washing oneself (not walking to washing place, the action itself) | | | | | | | | | | | | | | | 0 | 1 | | | | 2 | | | | 3 | | | 4 | Washing | |
| 3 | Toileting (not walking to toilet or constipation, the actions themselves) | | | | | | | | | | | | | | | 0 | 1 | | | | 2 | | | | 3 | | | 4 | Toileting | |
| 4 | Dressing oneself | | | | | | | | | | | | | | | 0 | 1 | | | | 2 | | | | 3 | | | 4 | Dressing | |
| 5 | Eating and drinking (not swallowing or preparing food, but the actions of feeding) | | | | | | | | | | | | | | | 0 | 1 | | | | 2 | | | | 3 | | | 4 | Eating | |
|  |  | | | | | | | | | | | | | | |  | | | | | | | | | | | | |  |  |
|  | **Over the past 6 months have you sought care from any of the following?** | | | | | | | | | | | | | | | | | | | | | | | | | | | |  |  |
|  | *(The earliest in calendar time is the first)* | | | | | | | | | | | | | | | Order of visiting | | | | | | | | | | | | |  |  |
| 6 | Traditional healer | | | | | | | | | | **Y** | | | | **N** |  | | | | | | | | | | | | | healer |  |
| 7 | Private practitioner | | | | | | | | | | **Y** | | | | **N** |  | | | | | | | | | | | | | practi |  |
| 8 | Hospital / Health centre / CHAM | | | | | | | | | | **Y** | | | | **N** |  | | | | | | | | | | | | | opd |  |
|  |  | | | | | | | | | | | | | | |  | | | | | | | | | | | | |  |  |
|  | **Anthropometry** | | | | | | | | | | | | | | |  | | | | | | | | | | | | |  |  |
| 9 | Height cm | | | | | | | | | | | | | | |  | | | | | | | | | | | | | height |  |
| 10 | Weight kg | | | | | | | | | | | | | | |  | | | | | | | | | | | | | weight |  |
| 11 | MUAC cm | | | | | | | | | | | | | | |  | | | | | | | | | | | | | muac |  |
| 12 | Interviewer Staff Code | | | | | | | | | | | | | | |  | | | | | | | | | | | | | rcdr |  |
|  |  | | | | | | | | | | | | | | |  | | | | | | | | | | | | |  |  |
|  | **Investigations** | | | | | | | | | | | | | | |  | | | | | | | | | | | | |  |  |
| 13 | Has the patient been bled for TLC and Hb today? | | | | | | | | | | | | | | | Y | | | | | | | N | | | | | | bled |  |
| 14 | Specimen Set number | | | | | | | | | | | | | | |  | | | | | | | | | | | | | specset |  |
| 15 | Staff Code | | | | | | | | | | | | | | |  | | | | | | | | | | | | |  |  |
|  | | | | | | | | | | | | | | |  | | | | | | | | | | | | |  |
|  |  | | | | | | | | | | | | | | |  | | | | | | | | | | | | |  |  |
|  | **Clinician’s Section** | | | | | | | | | | | | | | |  | | | | | | | | | | | | |  |  |
| 16 | Looking back, how long ago do you think you started to become sick from HIV? | | | | | | | | | | | | | | |  | | | | D | | | | M | | | Y | | sickdate |  |
| 17 | What were the main symptoms at that time? (list up to 3 symptoms and rank by severity) | | | | | | | | | | | | | | | rank | | | | | | |  | | | | | |  |  |
| 18 |  | | | | | | | | | | | | | | |  | | | | | | |  | | | | | | Sign1  Rank1 |  |
| 19 |  | | | | | | | | | | | | | | |  | | | | | | |  | | | | | | Sign2  Rank2 |  |
| 20 |  | | | | | | | | | | | | | | |  | | | | | | |  | | | | | | Sign3  Rank3 |  |
|  | **Findings*** | | | | | | | | | | | | | | |  | | | | | | | | | | | | |  |  |
| 21 | Prolonged fever (intermittent or constant >1 month) (history) (3) | | | | | | | | | | | | | | | Y | | | N | | | | | | | Unsure | | | fever |  |
| 22 | Chronic diarrhoea >1 month (history) (3) | | | | | | | | | | | | | | | Y | | | N | | | | | | | Unsure | | | diarhh |  |
| 23 | Unintentional weight loss (history) | | | | **S**evere (>10%) (3) | | | | **M**oderate (<10%) | | | | **N**one | | | S | | M | | | | N | | | | Unsure | | | wgtloss |  |
| 24 | Recurrent URTI (e.g. bacterial sinusitis) (history) (2) | | | | | | | | | | | | | | | Y | | | N | | | | | | | Unsure | | | urti |  |
| 25 | Herpes simplex infection, mucocutaneous >1 month or visceral (history) (4) | | | | | | | | | | | | | | | Y | | | N | | | | | | | Unsure | | | hsv |  |
| 26 | Herpes zoster (within the last 2 years) (history and exam) (2) | | | | | | | | | | | | | | | Y | | | N | | | | | | | Unsure | | | zoster |  |
| 27 | Pulmonary TB within the past 2 years (history) (3) | | | | | | | | | | | | | | | Y | | | N | | | | | | | Unsure | | | ptb |  |
| 28 | Extrapulmonary TB (history) (4) | | | | | | | | | | | | | | | Y | | | N | | | | | | | Unsure | | | etb |  |
|  |  | | | | | | | | | | | | | | |  | | |  | | | | | | |  | | |  |  |
| 29 | Generalised lymphadenopathy (nodes in more than one site) (exam) (1) | | | | | | | | | | | | | | | Y | | | N | | | | | | | Unsure | | | glap |  |
| 30 | Minor mucocutaneous manifestations (seborhoeic dermatitis, angular cheilitis, prurigo, fungal nails inf.) (exam) (2) | | | | | | | | | | | | | | | Y | | | N | | | | | | | Unsure | | | skin |  |
| 31 | HIV wasting syndrome (weight loss >10% with chronic fever or diarrhoea) (history) (4) | | | | | | | | | | | | | | | Y | | | N | | | | | | | Unsure | | | hivwast |  |
|  |  | | | | | | | | | | | | | | |  | | |  | | | | | | |  | | |  |  |
| 32 | Oral candidiasis (exam) (3) | | | | | | | | | | | | | | | Y | | | N | | | | | | | Unsure | | | oralcand |  |
| 33 | Candidiasis of oesophagus, trachea or bronchus (history) (4) | | | | | | | | | | | | | | | Y | | | N | | | | | | | Unsure | | | canddeep |  |
|  |  | | | | | | | | | | | | | | |  | | |  | | | | | | |  | | |  |  |
| 34 | Severe bacterial infections (e.g. pneumonia, pyomyositis) (history and exam) (3) | | | | | | | | | | | | | | | Y | | | N | | | | | | | Unsure | | | bactinf |  |
| 35 | Atypical mycobacteriosis, disseminated or lungs (diagnosed from cultured specimen) (4) | | | | | | | | | | | | | | | Y | | | N | | | | | | | Unsure | | | atmyc |  |
| 36 | Pneumocystis carinii pneumonia (diagnosed from CXR or clinically – response to high dose bactrim) (4) | | | | | | | | | | | | | | | Y | | | N | | | | | | | Unsure | | | pcp |  |
|  |  | | | | | | | | | | | | | | |  | | |  | | | | | | |  | | |  |  |
| 37 | Toxoplasmosis of the brain (mainly diagnosed through CT scan) (4) | | | | | | | | | | | | | | | Y | | | N | | | | | | | Unsure | | | toxo |  |
| 38 | Cryptococcal meningitis (diagnosed by LP –indian ink positive, or clinically) (4) | | | | | | | | | | | | | | | Y | | | N | | | | | | | Unsure | | | hivbrain |  |
| 39 | HIV encephalopathy (history and exam) (4) | | | | | | | | | | | | | | | Y | | | N | | | | | | | Unsure | | | hivbrain |  |
|  |  | | | | | | | | | | | | | | |  | | |  | | | | | | |  | | |  |  |
| 40 | Lymphoma (history and exam) (4) | | | | | | | | | | | | | | | Y | | | N | | | | | | | Unsure | | | lymp |  |
| 41 | Kaposi’s sarcoma (exam) (4) | | | | | | | | | | | | | | | Y | | | N | | | | | | | Unsure | | | ks |  |
|  |  | | | | | | | | | | | | | | |  | | | | | | | | | | | | |  |  |
